# 3-(3-Bromo­benz­yl)-1*H*-isochromen-1-one

**DOI:** 10.1107/S1600536809037246

**Published:** 2009-09-26

**Authors:** Farukh Iftakhar Ali, Tariq Mahmood Babar, Nasim Hasan Rama, Peter G. Jones

**Affiliations:** a Department of Chemistry, Quaid-i-Azam University, Islamabad 45320, Pakistan; b Institut für Anorganische und Analytische Chemie, Technische Universität Braunschweig, Postfach 3329, 38023 Braunschweig, Germany

## Abstract

In the title compound, C_16_H_11_BrO_2_, the isocoumarin ring system is planar (r.m.s. deviation = 0.015 Å) and subtends a dihedral angle of 88.90 (2)° with the bromo­benzene ring. In the crystal, mol­ecules are linked, forming a three-dimensional packing pattern involving C—H⋯O inter­actions, Br⋯O contacts [3.4734 (10) Å] and π–π stacking inter­actions with centroid–centroid distances ranging from 3.667 (2) to 3.765 (2) Å.

## Related literature

For the properties and applications of isocoumarins and 3,4-dihydro­isocoumarins, see: Chinworrungsee *et al.* (2002 ▶); Devienne *et al.* (2002 ▶); Mali & Babu (1998 ▶); Rama *et al.* (1998 ▶); Waters & Kozlowski (2001 ▶). For related structures, see: Abid *et al.* (2008 ▶); Babar *et al.* (2008 ▶).
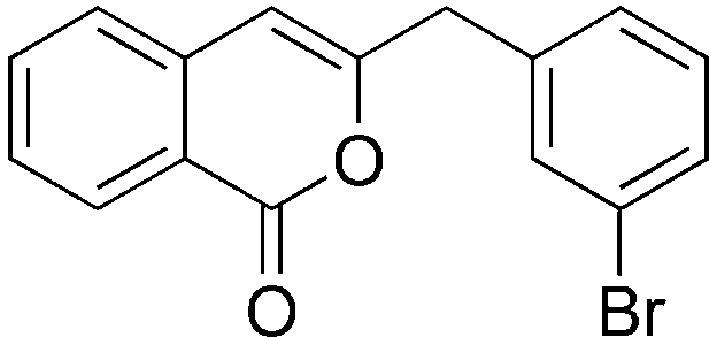


## Experimental

### 

#### Crystal data

C_16_H_11_BrO_2_
*M* *_r_* = 315.16
Triclinic, 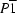

*a* = 7.4508 (5) Å
*b* = 8.1824 (6) Å
*c* = 11.3663 (8) Å
α = 90.130 (6)°
β = 98.392 (7)°
γ = 113.844 (8)°
*V* = 625.58 (8) Å^3^
*Z* = 2
Mo *K*α radiation
μ = 3.28 mm^−1^
*T* = 103 K
0.25 × 0.25 × 0.20 mm

#### Data collection

Oxford Xcalibur E diffractometer
Absorption correction: multi-scan (*CrysAlisPro*; Oxford Diffraction 2009 ▶) *T*_min_ = 0.922, *T*_max_ = 1.000
16250 measured reflections
3449 independent reflections
2899 reflections with *I* > 2σ(*I*)
*R*_int_ = 0.025

#### Refinement

*R*[*F* ^2^ > 2σ(*F* ^2^)] = 0.020
*wR*(*F* ^2^) = 0.045
*S* = 0.97
3449 reflections
172 parameters
H-atom parameters constrained
Δρ_max_ = 0.43 e Å^−3^
Δρ_min_ = −0.25 e Å^−3^

### 

Data collection: *CrysAlisPro* (Oxford Diffraction, 2009 ▶); cell refinement: *CrysAlisPro*; data reduction: *CrysAlisPro*; program(s) used to solve structure: *SHELXS97* (Sheldrick, 2008 ▶); program(s) used to refine structure: *SHELXL97* (Sheldrick, 2008 ▶); molecular graphics: *XP* (Siemens, 1994 ▶); software used to prepare material for publication: *SHELXL97*.

## Supplementary Material

Crystal structure: contains datablocks global, I. DOI: 10.1107/S1600536809037246/rz2357sup1.cif

Structure factors: contains datablocks I. DOI: 10.1107/S1600536809037246/rz2357Isup2.hkl

Additional supplementary materials:  crystallographic information; 3D view; checkCIF report

## Figures and Tables

**Table 1:** Hydrogen-bond geometry (Å, °)

*D*—H⋯*A*	*D*—H	H⋯*A*	*D*⋯*A*	*D*—H⋯*A*
C6—H6⋯O1^i^	0.95	2.58	3.4666 (16)	155
C10—H10*A*⋯O2^ii^	0.99	2.50	3.4685 (17)	166

Symmetry codes: (i) 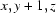; (ii) 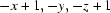.

**Fig. 1.**
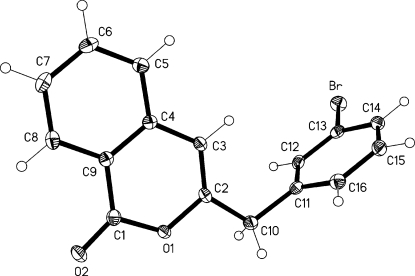
Molecular structure of the title compound showing the atom labelling scheme and displacement ellipsoids at the 50% probability level.

**Fig. 2.**
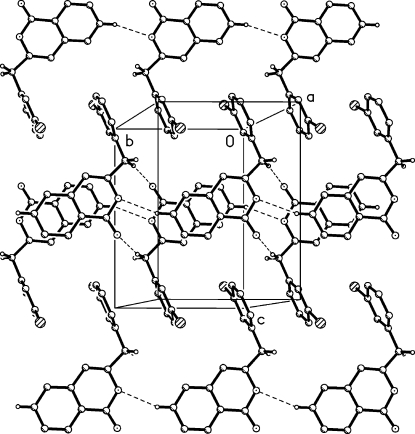
Packing diagram of the title compound viewed perpendicular to the bc plane. C—H···O hydrogen interactions are indicated by dashed lines. H atoms not involved in hydrogen bonding are omitted.

**Fig. 3.**
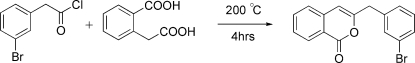
Reaction scheme.

## supplementary crystallographic information

### Comment

In recent years, there has been increasing interest in the synthesis of natural products, since they are an excellent and reliable source for the development of new drugs. Isocoumarins and 3,4-dihydroisocoumarins are a class of natural products that often occur as microbial metabolites and that have been found to exhibit interesting biological properties (Mali & Babu, 1998), including anti-fungal, anti-inflammatory, anti-allergic, antiangiogenic, anti-malaria (Chinworrungsee *et al.*, 2002), anti-bacterial (Rama *et al.*, 1998), anti-cancer, anti-virus (Waters & Kozlowski, 2001) and anti-microbial activities (Davienne *et al.*, 2002). In view of the importance of this class of compounds, the title compound, an isocoumarine derivative containing a 3-bromobenzyl substituent, has been synthesized and its crystal structure is reported here. We have previously reported the structures of the analogous fluorine and chlorine derivatives (Babar *et al.*, 2008; Abid *et al.*, 2008), which crystallize with two and three molecules respectively in the asymmetric unit.

The molecule of the title compound is shown in Fig. 1. The structure is not isotypic to either of the analogous derivatives. Bond lengths and angles may be regarded as normal by comparison with the earlier structures (Babar *et al.*, 2008; Abid *et al.*, 2008), although in each structure several bond angles are appreciably different from ideal values [*e. g.* in the current structure O2—C1—C9 126.19 (12), C3—C2—C10 128.78 (12), O1—C2—C10 109.56 (11), C2—C10—C11 112.98 (11)°]. The isocoumarin ring system and the bromobenzene ring are both planar within r.m.s. deviations of 0.015 Å and subtend a dihedral angle of 88.90 (2)°.

The packing diagram (Fig. 2) shows the molecules to be linked by two C—H···O hydrogen bonds (Table 1) and by π–π stacking interactions between the coumarin units and between the bromobenzene rings, with centroid-to-centroid distances ranging from 3.667 (2) to 3.765 (2) Å. A marginal Br···O interaction [Br···O2^i^ 3.4734 (10) Å; symmetry code: (i) -1 + *x*, *y*, -1 + *z*] is also observed.

### Experimental

A mixture of 2-(3-bromophenyl)acetic acid (5 g, 0.023 mol) and oxalyl chloride (2 ml, 0.024 mol) was stirred overnight. Completion of the reaction was indicated by cessation of gas evolution. Excess oxalyl chloride was removed under reduced pressure to afford 2-(3-bromophenyl)acetyl chloride. Homophthalic acid (1.0 g, 0.006 mol) was added and the solution was heated at 473 K for 4 h. The reaction mixture was dissolved in ethyl acetate and aqueous solution of sodium carbonate was added in order to remove the unreacted homophthalic acid. The organic layer was separated, concentrated and chromatographed on silica gel using pet ether as eluent to afford title compound (yield 65%,; m.p. 97–98°C) as a colourless solid. Crystals suitable for X-ray analysis were obtained by slow evaporation of an ethanol solution.

### Refinement

H atoms were placed in calculated positions and refined using a riding model with C—H = 0.95–0.99 Å and with *U*_iso_(H) = 1.2*U*_eq_(C).

### Crystal data

**Table:**

C_16_H_11_BrO_2_	*Z* = 2
*M**_r_* = 315.16	*F*(000) = 316
Triclinic, *P*1	*D*_x_ = 1.673 Mg m^−^^3^
Hall symbol: -P 1	Mo *K*α radiation, λ = 0.71073 Å
*a* = 7.4508 (5) Å	Cell parameters from 9750 reflections
*b* = 8.1824 (6) Å	θ = 2.7–30.7°
*c* = 11.3663 (8) Å	µ = 3.28 mm^−^^1^
α = 90.130 (6)°	*T* = 103 K
β = 98.392 (7)°	Block, colourless
γ = 113.844 (8)°	0.25 × 0.25 × 0.20 mm
*V* = 625.58 (8) Å^3^	

### Data collection

**Table:**

Oxford Xcalibur E diffractometer	3449 independent reflections
Radiation source: Enhance (Mo) X-ray Source	2899 reflections with *I* > 2σ(*I*)
graphite	*R*_int_ = 0.025
Detector resolution: 16.1419 pixels mm^-1^	θ_max_ = 29.6°, θ_min_ = 3.0°
ω scan	*h* = −10→10
Absorption correction: multi-scan (*CrysAlis PRO*; Oxford Diffraction 2009)	*k* = −11→11
*T*_min_ = 0.922, *T*_max_ = 1.000	*l* = −15→15
16250 measured reflections	

### Refinement

**Table:**

Refinement on *F*^2^	Primary atom site location: structure-invariant direct methods
Least-squares matrix: full	Secondary atom site location: difference Fourier map
*R*[*F*^2^ > 2σ(*F*^2^)] = 0.020	Hydrogen site location: inferred from neighbouring sites
*wR*(*F*^2^) = 0.045	H-atom parameters constrained
*S* = 0.97	*w* = 1/[σ^2^(*F*_o_^2^) + (0.0233*P*)^2^] where *P* = (*F*_o_^2^ + 2*F*_c_^2^)/3
3449 reflections	(Δ/σ)_max_ < 0.001
172 parameters	Δρ_max_ = 0.43 e Å^−^^3^
0 restraints	Δρ_min_ = −0.25 e Å^−^^3^

### Special details

**Table:**

Geometry. All e.s.d.'s (except the e.s.d. in the dihedral angle between two l.s. planes) are estimated using the full covariance matrix. The cell e.s.d.'s are taken into account individually in the estimation of e.s.d.'s in distances, angles and torsion angles; correlations between e.s.d.'s in cell parameters are only used when they are defined by crystal symmetry. An approximate (isotropic) treatment of cell e.s.d.'s is used for estimating e.s.d.'s involving l.s. planes.Non-bonded contact:3.4734 (0.0010) Br - O2_$5 157.19 (0.04) C13 - Br - O2_$5 144.68 (0.08) Br - O2_$5 - C1_$5 Operator for generating equivalent atoms: $5 *x* - 1, *y*, *z* - 1Least-squares planes (*x*,*y*,*z* in crystal coordinates) and deviations from them (* indicates atom used to define plane)- 1.8912 (0.0020) *x* + 7.3803 (0.0014) *y* + 4.4053 (0.0037) *z* = 0.7728 (0.0010)* 0.0088 (0.0008) C10 * -0.0063 (0.0011) C11 * 0.0058 (0.0010) C12 * 0.0167 (0.0011) C13 * 0.0215 (0.0010) C14 * -0.0036 (0.0009) C15 * -0.0224 (0.0011) C16 * -0.0204 (0.0006) BrRms deviation of fitted atoms = 0.01517.0134 (0.0011) *x* - 0.6456 (0.0018) *y* - 2.6242 (0.0032) *z* = 3.6495 (0.0018)Angle to previous plane (with approximate e.s.d.) = 88.90 (0.02)* -0.0183 (0.0009) C10 * -0.0095 (0.0009) O1 * 0.0027 (0.0011) C1 * -0.0081 (0.0008) O2 * 0.0273 (0.0011) C3 * 0.0129 (0.0012) C4 * -0.0052 (0.0011) C5 * -0.0241 (0.0011) C6 * -0.0078 (0.0012) C7 * 0.0144 (0.0012) C8 * 0.0159 (0.0012) C9Rms deviation of fitted atoms = 0.0152
Refinement. Refinement of *F*^2^ against ALL reflections. The weighted *R*-factor *wR* and goodness of fit *S* are based on *F*^2^, conventional *R*-factors *R* are based on *F*, with *F* set to zero for negative *F*^2^. The threshold expression of *F*^2^ > σ(*F*^2^) is used only for calculating *R*-factors(gt) *etc*. and is not relevant to the choice of reflections for refinement. *R*-factors based on *F*^2^ are statistically about twice as large as those based on *F*, and *R*- factors based on ALL data will be even larger.

### Fractional atomic coordinates and isotropic or equivalent isotropic displacement parameters (Å^2^)

**Table:**

	*x*	*y*	*z*	*U*_iso_*/*U*_eq_	
Br	0.13942 (2)	0.205557 (19)	−0.113746 (13)	0.02023 (5)	
O1	0.70881 (14)	0.19099 (12)	0.46026 (8)	0.0146 (2)	
O2	0.78538 (15)	0.22208 (13)	0.65672 (8)	0.0195 (2)	
C1	0.75969 (19)	0.29281 (17)	0.56653 (12)	0.0141 (3)	
C2	0.67678 (19)	0.25762 (17)	0.35091 (11)	0.0128 (3)	
C3	0.69140 (19)	0.42394 (17)	0.34239 (11)	0.0136 (3)	
H3	0.6699	0.4662	0.2661	0.016*	
C4	0.73964 (18)	0.54146 (17)	0.44789 (11)	0.0124 (3)	
C5	0.7521 (2)	0.71742 (18)	0.44484 (12)	0.0170 (3)	
H5	0.7288	0.7643	0.3706	0.020*	
C6	0.7982 (2)	0.82214 (18)	0.54931 (13)	0.0182 (3)	
H6	0.8045	0.9404	0.5465	0.022*	
C7	0.8354 (2)	0.75669 (19)	0.65887 (13)	0.0185 (3)	
H7	0.8683	0.8306	0.7301	0.022*	
C8	0.8248 (2)	0.58509 (18)	0.66416 (12)	0.0162 (3)	
H8	0.8507	0.5406	0.7389	0.019*	
C9	0.77565 (19)	0.47639 (17)	0.55899 (11)	0.0126 (3)	
C10	0.6231 (2)	0.11432 (17)	0.25339 (11)	0.0161 (3)	
H10A	0.4972	0.0138	0.2639	0.019*	
H10B	0.7280	0.0682	0.2602	0.019*	
C11	0.5989 (2)	0.17984 (17)	0.12982 (11)	0.0144 (3)	
C12	0.4136 (2)	0.16677 (17)	0.07491 (11)	0.0146 (3)	
H12	0.3011	0.1147	0.1140	0.018*	
C13	0.39414 (19)	0.23009 (17)	−0.03706 (12)	0.0143 (3)	
C14	0.5553 (2)	0.30743 (18)	−0.09635 (12)	0.0155 (3)	
H14	0.5401	0.3518	−0.1726	0.019*	
C15	0.7391 (2)	0.31864 (17)	−0.04193 (12)	0.0165 (3)	
H15	0.8510	0.3704	−0.0815	0.020*	
C16	0.7613 (2)	0.25498 (17)	0.06999 (12)	0.0157 (3)	
H16	0.8880	0.2627	0.1061	0.019*	

### Atomic displacement parameters (Å^2^)

**Table:**

	*U*^11^	*U*^22^	*U*^33^	*U*^12^	*U*^13^	*U*^23^
Br	0.01563 (7)	0.02239 (8)	0.02281 (8)	0.00845 (6)	0.00152 (5)	0.00243 (6)
O1	0.0220 (5)	0.0115 (5)	0.0106 (5)	0.0075 (4)	0.0015 (4)	0.0015 (4)
O2	0.0273 (6)	0.0183 (5)	0.0130 (5)	0.0098 (5)	0.0022 (4)	0.0048 (4)
C1	0.0134 (6)	0.0152 (7)	0.0135 (7)	0.0051 (6)	0.0033 (5)	0.0009 (5)
C2	0.0130 (6)	0.0147 (6)	0.0106 (6)	0.0051 (5)	0.0027 (5)	0.0033 (5)
C3	0.0162 (7)	0.0141 (7)	0.0107 (6)	0.0065 (6)	0.0016 (5)	0.0022 (5)
C4	0.0109 (6)	0.0123 (6)	0.0145 (7)	0.0046 (5)	0.0033 (5)	0.0014 (5)
C5	0.0191 (7)	0.0163 (7)	0.0170 (7)	0.0087 (6)	0.0031 (5)	0.0034 (6)
C6	0.0188 (7)	0.0124 (7)	0.0246 (8)	0.0069 (6)	0.0053 (6)	−0.0005 (6)
C7	0.0181 (7)	0.0183 (7)	0.0183 (7)	0.0062 (6)	0.0045 (6)	−0.0049 (6)
C8	0.0164 (7)	0.0187 (7)	0.0123 (7)	0.0054 (6)	0.0039 (5)	0.0009 (5)
C9	0.0111 (6)	0.0130 (6)	0.0138 (7)	0.0045 (5)	0.0034 (5)	0.0013 (5)
C10	0.0218 (7)	0.0117 (6)	0.0141 (7)	0.0060 (6)	0.0028 (5)	0.0010 (5)
C11	0.0208 (7)	0.0089 (6)	0.0124 (7)	0.0053 (6)	0.0014 (5)	−0.0030 (5)
C12	0.0173 (7)	0.0119 (6)	0.0140 (7)	0.0046 (6)	0.0052 (5)	−0.0014 (5)
C13	0.0147 (6)	0.0121 (6)	0.0155 (7)	0.0058 (5)	0.0001 (5)	−0.0032 (5)
C14	0.0196 (7)	0.0137 (7)	0.0131 (7)	0.0068 (6)	0.0021 (5)	0.0004 (5)
C15	0.0170 (7)	0.0149 (7)	0.0170 (7)	0.0051 (6)	0.0055 (5)	0.0019 (6)
C16	0.0159 (7)	0.0145 (7)	0.0154 (7)	0.0057 (6)	0.0002 (5)	−0.0012 (5)

### Geometric parameters (Å, °)

**Table:**

Br—C13	1.9001 (13)	C11—C16	1.3947 (19)
O1—C1	1.3796 (15)	C12—C13	1.3854 (18)
O1—C2	1.3859 (15)	C13—C14	1.3859 (19)
O2—C1	1.2073 (15)	C14—C15	1.3842 (18)
C1—C9	1.4618 (18)	C15—C16	1.3887 (18)
C2—C3	1.3256 (18)	C3—H3	0.9500
C2—C10	1.4996 (18)	C5—H5	0.9500
C3—C4	1.4426 (18)	C6—H6	0.9500
C4—C9	1.4035 (18)	C7—H7	0.9500
C4—C5	1.4056 (18)	C8—H8	0.9500
C5—C6	1.3787 (19)	C10—H10A	0.9900
C6—C7	1.392 (2)	C10—H10B	0.9900
C7—C8	1.3759 (19)	C12—H12	0.9500
C8—C9	1.3994 (18)	C14—H14	0.9500
C10—C11	1.5166 (18)	C15—H15	0.9500
C11—C12	1.3911 (18)	C16—H16	0.9500
			
C1—O1—C2	122.56 (10)	C15—C14—C13	118.52 (13)
O2—C1—O1	117.22 (12)	C14—C15—C16	120.63 (13)
O2—C1—C9	126.19 (12)	C15—C16—C11	120.46 (13)
O1—C1—C9	116.59 (11)	C2—C3—H3	119.7
C3—C2—O1	121.65 (12)	C4—C3—H3	119.7
C3—C2—C10	128.78 (12)	C6—C5—H5	119.9
O1—C2—C10	109.56 (11)	C4—C5—H5	119.9
C2—C3—C4	120.62 (12)	C5—C6—H6	119.6
C9—C4—C5	118.50 (12)	C7—C6—H6	119.6
C9—C4—C3	118.22 (12)	C8—C7—H7	119.9
C5—C4—C3	123.28 (12)	C6—C7—H7	119.9
C6—C5—C4	120.19 (13)	C7—C8—H8	120.1
C5—C6—C7	120.77 (13)	C9—C8—H8	120.1
C8—C7—C6	120.14 (13)	C2—C10—H10A	109.0
C7—C8—C9	119.74 (13)	C11—C10—H10A	109.0
C8—C9—C4	120.65 (12)	C2—C10—H10B	109.0
C8—C9—C1	119.01 (12)	C11—C10—H10B	109.0
C4—C9—C1	120.34 (12)	H10A—C10—H10B	107.8
C2—C10—C11	112.98 (11)	C13—C12—H12	120.2
C12—C11—C16	119.06 (12)	C11—C12—H12	120.2
C12—C11—C10	120.17 (12)	C15—C14—H14	120.7
C16—C11—C10	120.77 (12)	C13—C14—H14	120.7
C13—C12—C11	119.64 (12)	C14—C15—H15	119.7
C12—C13—C14	121.67 (13)	C16—C15—H15	119.7
C12—C13—Br	119.50 (10)	C15—C16—H16	119.8
C14—C13—Br	118.81 (10)	C11—C16—H16	119.8
			
C2—O1—C1—O2	−179.57 (11)	O2—C1—C9—C8	−0.1 (2)
C2—O1—C1—C9	0.95 (17)	O1—C1—C9—C8	179.34 (11)
C1—O1—C2—C3	−0.72 (19)	O2—C1—C9—C4	−179.30 (13)
C1—O1—C2—C10	−179.69 (11)	O1—C1—C9—C4	0.14 (18)
O1—C2—C3—C4	−0.6 (2)	C3—C2—C10—C11	5.0 (2)
C10—C2—C3—C4	178.12 (13)	O1—C2—C10—C11	−176.15 (11)
C2—C3—C4—C9	1.66 (19)	C2—C10—C11—C12	−90.92 (15)
C2—C3—C4—C5	−178.28 (13)	C2—C10—C11—C16	88.60 (15)
C9—C4—C5—C6	−0.22 (19)	C16—C11—C12—C13	−0.72 (19)
C3—C4—C5—C6	179.72 (13)	C10—C11—C12—C13	178.81 (11)
C4—C5—C6—C7	0.9 (2)	C11—C12—C13—C14	−0.27 (19)
C5—C6—C7—C8	−0.6 (2)	C11—C12—C13—Br	178.14 (9)
C6—C7—C8—C9	−0.2 (2)	C12—C13—C14—C15	0.9 (2)
C7—C8—C9—C4	0.9 (2)	Br—C13—C14—C15	−177.54 (10)
C7—C8—C9—C1	−178.34 (13)	C13—C14—C15—C16	−0.50 (19)
C5—C4—C9—C8	−0.63 (19)	C14—C15—C16—C11	−0.5 (2)
C3—C4—C9—C8	179.43 (12)	C12—C11—C16—C15	1.09 (19)
C5—C4—C9—C1	178.56 (12)	C10—C11—C16—C15	−178.44 (12)
C3—C4—C9—C1	−1.38 (18)		

### Hydrogen-bond geometry (Å, °)

**Table:**

*D*—H···*A*	*D*—H	H···*A*	*D*···*A*	*D*—H···*A*
C6—H6···O1^i^	0.95	2.58	3.4666 (16)	155
C10—H10A···O2^ii^	0.99	2.50	3.4685 (17)	166

Symmetry codes: (i) *x*, *y*+1, *z*; (ii) −*x*+1, −*y*, −*z*+1.
